# A223 ORBITAL MYOSITIS AS THE INITIAL PRESENTING SIGN OF CROHN’S DISEASE IN AN ADOLESCENT FEMALE

**DOI:** 10.1093/jcag/gwae059.223

**Published:** 2025-02-10

**Authors:** N AlAyedh, a alhadab, T Cellucci, M Batthish, S Heede, M Zachos

**Affiliations:** Pediatrics, McMaster Children’s Hospital, Hamilton, ON, Canada; Pediatrics, McMaster Children’s Hospital, Hamilton, ON, Canada; Pediatrics, McMaster Children’s Hospital, Hamilton, ON, Canada; Pediatrics, McMaster Children’s Hospital, Hamilton, ON, Canada; Pediatrics, McMaster Children’s Hospital, Hamilton, ON, Canada; Pediatrics, McMaster Children’s Hospital, Hamilton, ON, Canada

## Abstract

**Background:**

Ocular extraintestinal manifestations (O-EIM) of inflammatory bowel disease (IBD) are rare, with most being attributed to scleritis, episcleritis and uveitis. Orbital myositis, however, is an exceedingly rare manifestation that is described as acute or chronic inflammation of one or more extraocular muscles leading to symptoms related to the mass effect including orbital pain, swelling, ophthalmoplegia, proptosis, and diplopia. To date, 3 reports of varying ocular myositis preceding IBD diagnosis have been described in pediatrics.

**Aims:**

This report describes a case of unilateral orbital myositis, which was the initial and only symptom of newly diagnosed adolescent with Crohn’s disease (CD).

**Methods:**

A chart review was conducted and placed into the context of this rare presentation.

**Results:**

15-year-old female presented with acute 6^th^ cranial nerve palsy with ipsilateral optic disc edema, raising concern for raised intracranial pressure. Magnetic resonance imaging (MRI) revealed a retro-orbital mass on the left lateral rectus muscle, consistent with orbital pseudo tumor and myositis, no intracranial tumor or vascular lesion. She was treated with 3-month course of prednisone. An inflammatory condition was suspected based on the unusual presentation of orbital myositis which responded well to prednisone. Alternative infectious or rheumatologic causes of orbital pseudotumor were excluded and the patient was treated with corticosteroids prior to gastroenterology (GI) consultation. She was found to have elevated C-reactive protein (CRP), hypoalbuminemia and anemia at presentation. Fecal calprotectin (649)) was subsequently found to be positive at GI consultation after corticosteroid treatment initiation. Given the patient did not exhibit any GI signs or symptoms at the time of the evaluation, further GI investigations were deferred to after completion of steroids. Fecal calprotectin was persistently elevated (1463) after resolution of ocular symptoms and completion of steroids. Magnetic resonance enterography (MRE) identified inflammation of the terminal ileum. A definitive diagnosis of CD was established following upper GI endoscopy and colonoscopy with biopsies.

**Conclusions:**

Orbital myositis is a rare ocular manifestation of CD. Early recognition and treatment are critical as they may precede the development of overt gastrointestinal symptoms, potentially delaying diagnosis if not considered. Given the rarity of this presentation, further studies are needed to better understand the pathophysiological link between CD and orbital myositis.

**Funding Agencies:**

None

